# ^1^H-MAS-NMR Chemical Shifts in Hydrogen-Bonded Complexes of Chlorophenols (Pentachlorophenol, 2,4,6-Trichlorophenol, 2,6-Dichlorophenol, 3,5-Dichlorophenol, and *p*-Chlorophenol) and Amine, and H/D Isotope Effects on ^1^H-MAS-NMR Spectra

**DOI:** 10.3390/molecules18044786

**Published:** 2013-04-22

**Authors:** Hisashi Honda

**Affiliations:** Graduate School of Nanobioscience, Yokohama City University, Kanazawa-ku, Yokohama 236-0027, Japan; E-Mail: hhonda@yokohama-cu.ac.jp; Tel.: +81-45-787-2393; Fax: +81-45-787-2413

**Keywords:** hydrogen bond, ^1^H-MAS-NMR, H/D isotope effect

## Abstract

Chemical shifts (CS) of the ^1^H nucleus in N···H···O type hydrogen bonds (H-bond) were observed in some complexes between chlorophenols [pentachlorophenol (PCP), 2,4,6-tricholorophenol (TCP), 2,6-dichlorophenol (26DCP), 3,5-dichlorophenol (35DCP), and *p*-chlorophenol (*p*CP)] and nitrogen-base (N-Base) by solid-state high-resolution ^1^H-NMR with the magic-angle-spinning (MAS) method. Employing N-Bases with a wide range of p*K*_a_ values (0.65–10.75), ^1^H-MAS-NMR CS values of bridging H atoms in H-bonds were obtained as a function of the N-Base’s p*K*_a_. The result showed that the CS values were increased with increasing p*K*_a_ values in a range of Δp*K*_a_ < 0 [Δp*K*_a_ = p*K*_a_(N-Base) - p*K*_a_(chlorophenols)] and decreased when Δp*K*_a_ > 2: The maximum CS values was recorded in the PCP (p*K*_a_ = 5.26)–4-methylpyridine (6.03), TCP (6.59)–imidazole (6.99), 26DCP (7.02)–2-amino-4-methylpyridine (7.38), 35DCP (8.04)–4-dimethylaminopyridine (9.61), and *p*CP (9.47)–4-dimethylaminopyridine (9.61) complexes. The largest CS value of 18.6 ppm was recorded in TCP–imidazole crystals. In addition, H/D isotope effects on ^1^H-MAS-NMR spectra were observed in PCP–2-amino-3-methylpyridine. Based on the results of CS simulation using a B3LYP/6-311+G** function, it can be explained that a little changes of the N–H length in H-bond contribute to the H/D isotope shift of the ^1^H-MAS-NMR peaks.

## 1. Introduction

X-ray diffraction (XRD) measurements have been used to detect A···B length changes by deuterium substitution in A-H···B type H-bonds [1,2,3,4]. Determining the accurate position of H atoms, however, has been difficult by this method. Nuclear quadrupole resonance (NQR) methods are often employed to investigate H/D isotope effects. ^35^Cl NQR measurements have shown frequency shifts of dozens of kHz for covalently-attached Cl atoms and up to several hundred kHz (occasionally reaching MHz order) for ionic Cl^−^ atoms by deuterium substitution [5,6,7,8,9,10,11,12,13,14,15,16]. In a case of piperidinium and pyrrolidinium *p*-chlorobenzoate crystals, large ^35^Cl NQR frequency shifts of *ca*. 300 kHz have been detected by deuterium substitution of H atoms forming H-bonds, although the Cl atom doesn’t contribute to H-bonds in the crystals [17,18]. In contrast, ^79^Br NQR have exhibited small H/D shifts in piperidinium and pyrrolidinium *p*-bromobenzoate solids [19], despite the fact that these *p*-chloro-benzoate and *p*-bromobenzoate have the similar crystal structures [17,18,20,21]. Nuclear magnetic resonance (NMR) measurements have been sometimes used to detect electron density changes of constructive molecules upon deuteration. ^13^C-CP/MAS-NMR spectra (CP: cross polarization, MAS: magic-angle-spinning) have been employed to study H/D isotope effects [22,23,24,25,26], however, such lines generally show slight shift after deuterium substitution. ^1^H-MAS-NMR spectra of piperidinium and pyrrolidinium *p*-chlorobenzoate have displayed significant envelope changes by deuterium substitution, while the ^13^C-CP/MAS-NMR spectra show only small changes [18,27]. In these crystals, small changes of molecular arrangements by deuteration contribute to anomalous H/D isotope shift of the ^1^H-MAS-NMR spectra and ^35^Cl NQR frequencies. In the case of piperidinium and pyrrolidinium *p*-bromobenzoate, small H/D isotope effects on the ^1^H-MAS-NMR spectral lines are reported [19].

In a case of pentachlorophenol (abbreviated to PCP) complexes, it has been reported that PCP forms salts with nitrogen-bases (N-Base) covering a broad p*K*_a_ range (0.65–11.20). These crystals have been employed to detect proton-transfer equilibrium between O–H···N (covalent form) and O^−^···H–N^+^ (ionic form) [28,29,30,31,32,33,34,35,36,37,38,39,40,41,42,43,44,45]. ^35^Cl NQR frequency measurements have reported that the NQR frequencies give constant values of *ca*. 37.6 MHz in the small p*K*_a_ ranges of N-Base, and successively decreased with increasing p*K*_a_ values in the middle p*K*_a_ range of 5–7, and take a constant of 36.9 MHz in p*K*_a_ > 7 [28]. An inversion point of the frequency slope is shown at around p*K*_a_ of 6. In addition, large H/D isotope shift of 250 kHz is detected in ^35^Cl NQR frequencies of PCP–4-methylpyridine (4MP; p*K*_a_ = 6.06). In contrast, other PCP–N-Base complexes show little H/D isotope shifts [12]. These investigations have expected to proton transfer exhibiting in the PCP-4MP complex, and XRD and neutron diffraction measurements have revealed proton transfers between O–H···N and O^−^···H-N^+^ states [29,30,31,32,33,34,35,36]. In addition, it has been reported that PCP and 4MP molecules are linked by the strongest known intermolecular O··H··N type H-bond in solids [33]. This crystal (triclinic) changes to a monoclinic structure exhibiting a weak H-bond after deuteration of the H-bond. The origin of the isotopic polymorphism is explained by dipole moment changes [33]: Since the O–D length is shorter than the O-H separation, the O···N distance becomes long upon deuteration. This change effects onto the local dipole moment and the dipole−dipole interaction between adjacent coupled H-bonds is reduced. 

In contrast of PCP–4MP, a few studies of PCP complexes with other N-Bases have been reported [41,42,43,44,45], and are rare for 2,4,6-trichlorophenol (TCP), 2,6-dichlorophenol (26DCP), 3,5-dichlorophenaol (35DCP), and *p*-chlorophenol (*p*CP) complexes with N-Bases. In the present study, ^1^H-MAS-NMR spectra were observed in PCP, TCP, 26DCP, 35DCP, and *p*CP complexes with N-Bases listed in Table 1 (left column), and in order to detect H/D isotope effects on ^1^H-MAS-NMR spectra, some deuterium substituted salts in which H atoms contributing H-bond was exchanged by D atoms were introduced. 

**Table 1:** ^1^H chemical shifts (ppm) of bridging H atoms observed in phenols and N-Bases complexes. Here, p*K*_a_ values [28] of phenols and N-Bases are shown in parenthesis.

		Pentachloro- phenol	2,4,6-Trichloro phenol	2,6-Dichloro phenol	3,5-Dichloro phenol	*p*-Chloro phenol
	Symbols	PCP (5.26)	TCP (6.59)	26DCP (7.02)	35DCP (8.04)	*p*-CP (9.47)
Pyridine	PYR (0.65)	10.85	9.20	8.31	8.00	9.80
3-Cyanopyridine	3CP (1.35)	12.18				
4-Cyanopyridine	4CP (1.86)	11.40	9.63	9.14	9.20	10.80
3-Bromopyridine	3BP (2.85)	12.54	9.87			
4-Acethylpyridine	4AP (12.02)	12.02			11.51	12.59
Quinoline	QL (14.11)	14.11	11.13			
Isoquinoline	IQL (5.40)	13.35				
2-Methylpyridine	2MP (5.94)	15.49, 12.69				
4-Methylpyridine	4MP (6.03)	17.85, 12.91				
Imidazole	IMID (16.92)	16.92	18.62, 15.84			
2-Amino-3-methyl-pyridine	2A3MP (7.21)	16.10			13.97	
2-Amino-4-methyl-pyridine	2A4MP (7.38)		15.06	15.71, 9.77		14.14
Triethylenediamine	TEDA (8.82)	14.36	13.73	12.74	14.05	11.90
4-Dimethlamino-pyridine	4DMAP (9.61)	13.18			15.64	14.29
Triethylamine	TEA (10.75)	13.11, 10.49		10.40		

## 2. Results and Discussion

### 2.1. pK_a_ Dependences of ^1^H-MAS-NMR Chemical Shift

**Figure 1:**
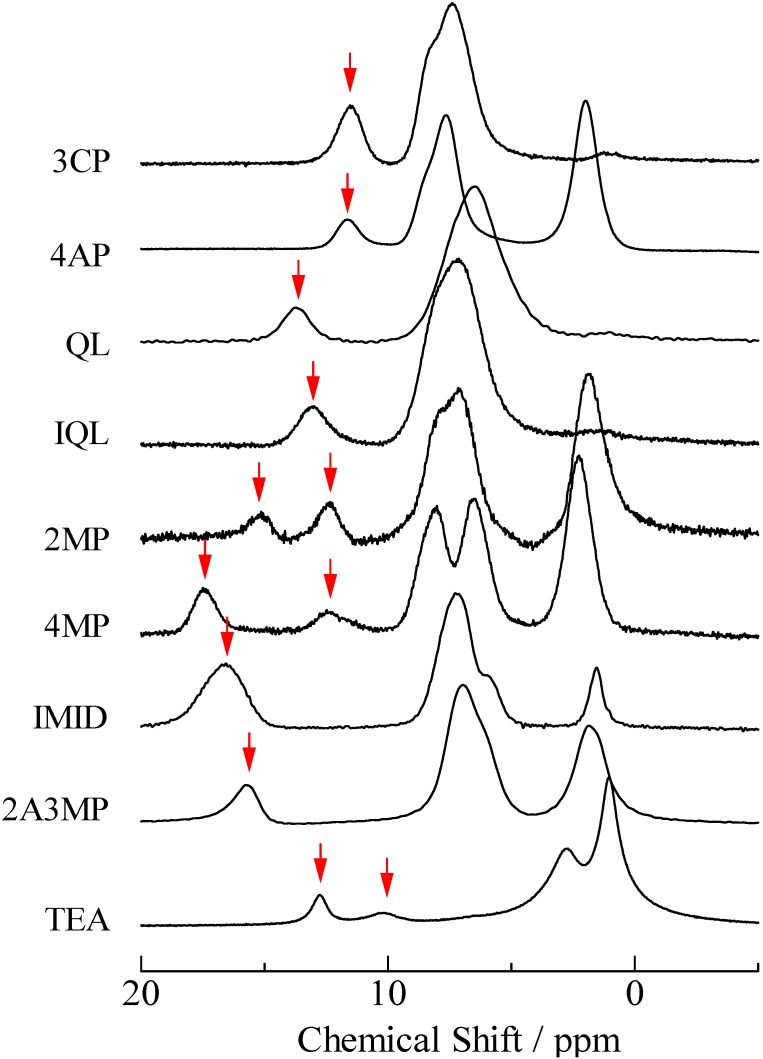
^1^H-MAS-NMR spectra of pentachlorophenol complexes. Peaks of bridging H atoms are shown with arrows.

^1^H-MAS-NMR spectra obtained for the PCP complexes are shown in Figure 1. The peaks obtained in a range of 9 to 18 ppm could be assigned to the H atom in O···H···N type H-bonds. This assignment could be supported by comparing with results of DFT calculation using a B3LYP/6-311++G** function. The ^1^H-MAS-NMR spectra show two results: (i) the CS values of bridging H atoms were increased and gradually decreased with increasing p*K*_a_ values of N-Bases. (ii) Two peaks of the bridging H atom were recorded in PCP–2MP, PCP–4MP, PCP–TEA.

**Figure 2:**
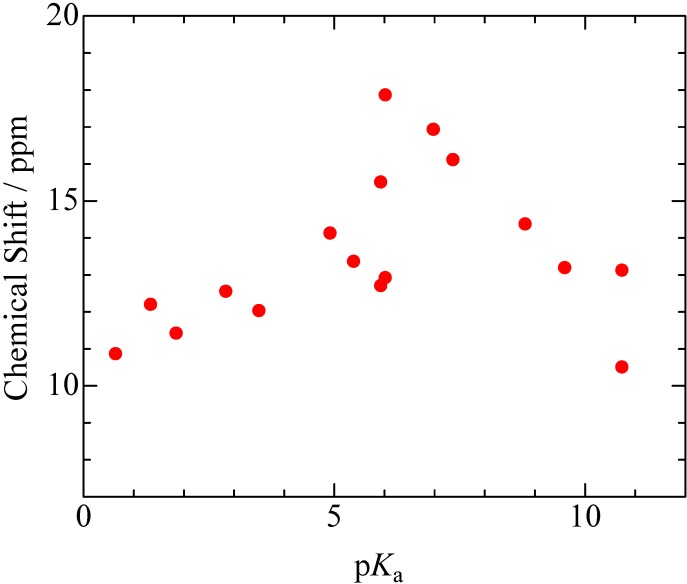
^1^H-MAS-NMR chemical shifts of pentachlorophenol complexes plotted as a function of N-Base’s p*K*_a_.

In order to discuss the result (i), the ^1^H-NMR CS values of the bridging H atom are plotted as a function of N-Base’s p*K*_a_ (Figure 2). This figure reveals that a maximum value of CS is found in PCP–4MP complex; the p*K*_a_ value of 4MP (6.03) is slightly larger than that of PCP (5.26). This p*K*_a_ value of 6.03 is agreement in the inversion point reported in the ^35^Cl NQR frequency curve [28,29]: It has been shown that the ^35^Cl NQR frequencies of the PCP complexes have a constant value of *ca*. 37.6 MHz in the range of p*K*_a_ < 5, and successively decreased with increasing p*K*_a_ in the middle p*K*_a_ range of 5–7, and take a constant value of *ca*. 36.9 MHz in p*K*_a_ > 7. The different dependence of ^1^H-NMR CS values and ^35^Cl NQR frequencies can be explained that the former method can directly detect the electron density of the bridging H atom, in contrast, the later method can estimate ionicity of the PCP molecule. Based on the previous reports [28,29,30,31,32,33,34,35,36,37], the neutralizing state of PCP (C_6_Cl_5_OH) is obtained in the range of p*K*_a_ < 5 and the anion form (C_6_Cl_5_O^−^) is detected in the high p*K*_a_ ranges; in the middle p*K*_a_ ranges, the proton transfer between PCP and N-Bases is suggested. Since a CS value of ^1^H nucleus can be theoretically considered as a function of charge density (the higher positive-charge results in the larger CS value, because ^1^H CS values are mainly determined by diamagnetic terms rather than paramagnetic ones), it can be concluded that the most positive-charge of the bridging H atom is recorded in the 4MP salt in the PCP complexes. Increasing the p*K*_a_ values from 6.03, the CS values are gradually decreased. Based on results of ^35^Cl NQR measurements [28], average position of the bridging H atom is shifted from PCP to N-Base compounds with increasing N-Base’s p*K*_a_. The result of decreasing ^1^H-NMR CS values suggests that positive charge of the H atom is decreased in the range of p*K*_a_ > 6.03: This result can be considered that positive charge of the bridging H atom is delocalized onto N-Base molecules with increasing the p*K*_a_ values, because the longer O–H distance results in the shorter H-N separation. 

**Figure 3:**
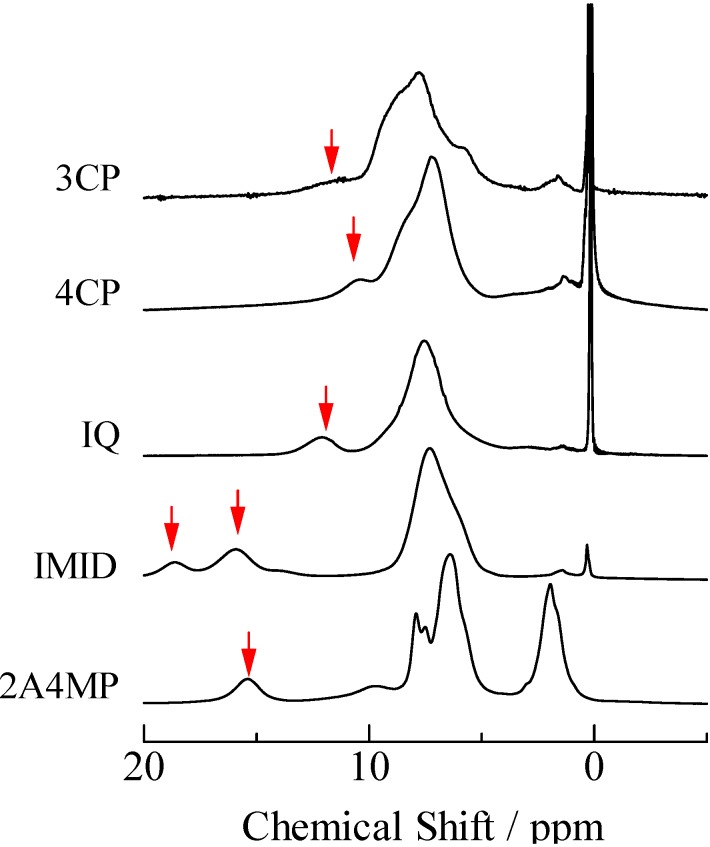
^1^H-MAS-NMR spectra of 2,4,6-trichlorophenol complexes. The signal of inner reference of silicon rubber was recorded at 0.12 ppm. Peaks of bridging H atoms are shown with arrows.

**Figure 4:**
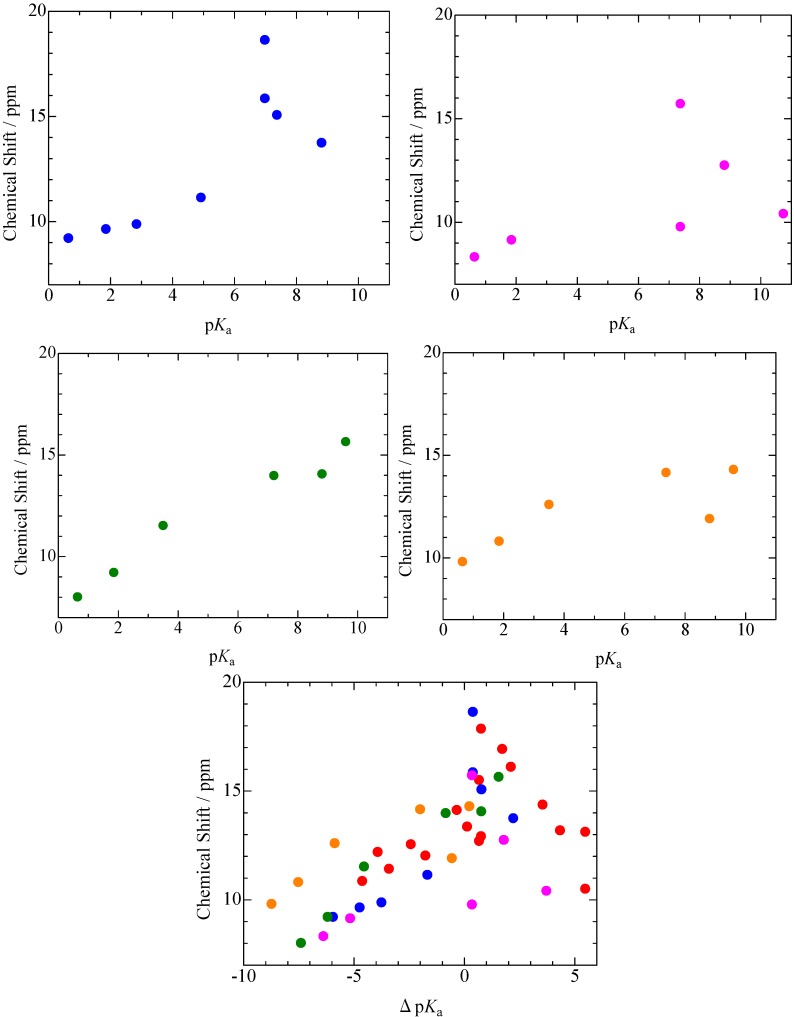
^1^H-MAS-NMR spectra of 2,4,6-trichlorophenol(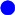), 2,6-dichlorophenol(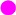), 3,5-dichlorophenol(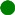), and *p*-chlorophenol(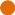) complexes plotted as a function of p*K*_a_ and Δp*K*_a_ = p*K*_a_ (N-Bases) - p*K*_a_ (chlorophenols) (pentachlorophenol(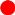)).

In the case of TCP complexes, ^1^H-MAS-NMR spectra as shown in Figure 3 were obtained. Based on results of CS simulation using the same function as described above, it could be assigned that the peaks observed around 5–9 ppm were superimposed by the H atoms of TCP and N-Base. The CS values assigned to the bridging H atom of TCP, 26DCP, 35DCP, and *p*CP complexes (the signals were recorded in a range of 9–20 ppm) are summarized in Figure 4. In this figure, the CS values of PCP complexes are also displayed against Δp*K*_a_ which is defined by p*K*_a_(N-Base) - p*K*_a_(chlorophenols). This figure suggests that CS values of the bridging H atoms are correlated with Δp*K*_a_ and the maximum CS value of each complex is recorded at Δp*K*_a_ of *ca*. 1. In addition, the largest CS value of 18.6 ppm was recorded in TCP–IMID (in the case of PCP–4MP, the CS value of 17.8 ppm was obtained). Based on the previous reports about PCP–4MP [12,28,29,30,31,32,33,34,35,36], it can be considered that TCP and IMID molecules are linked by very strong H-bond and proton transfer can be also expected in the crystal.

**Figure 5:**
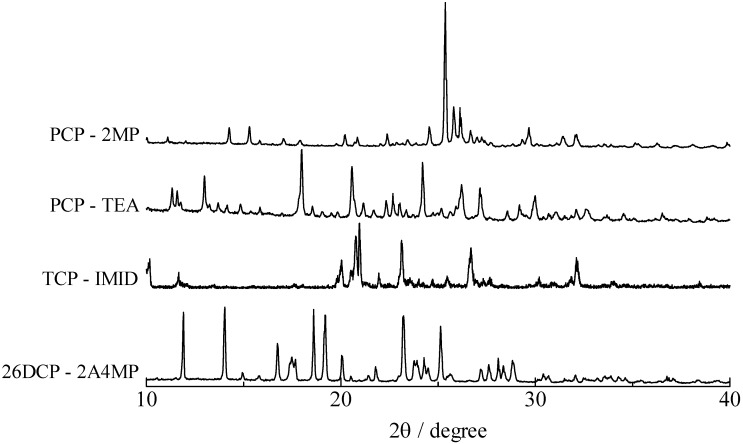
XRD spectra of pentachlorophenol–2-methylpyridine (PCP–2MP), pentachlorophenol–triethylamine (PCP–TEA), 2,4,6-trichlorophenol–imidazole (TCP–IMID), and 2,6-dichlorophenol–2-amino-4-methylpyridine (26DCP–2A4MP) complexes.

Two ^1^H-MAS-NMR peaks of the bridging H atom were recorded in PCP–2MP, PCP–4MP, and PCP–TEA complexes as described above [result (ii)]. The same result is detected in TCP–IMID, and 26DCP–2A4MP salts. In the case of the 4MP complex, two signals are detected at 12.9 and 17.8 ppm. This line shape is similar to the reported envelope [33,34]. These literatures show that there are two kinds of crystallographic structure (monoclinic and triclinic) in the PCP–4MP solids. Based on the reports, the peaks observed at 12.9 and 17.8 ppm can be assigned to the bridging H atom in the monoclinic and triclinic forms, respectively. Based on the result, it could be considered that the other complexes of PCP–2MP, PCP–TEA TCP–IMID, and 26DCP–2A4MP also have two kinds of crystal forms. In order to confirm this expectation, X-ray diffraction (XRD), ^13^C-CP/MAS-NMR, and temperature dependences of ^1^H-MAS-NMR spectra measurements were carried out. The results of XRD powdered patterns observed in them are displayed in Figure 5. In the case of PCP–2MP, the space group of P1 has been shown [41]. Subtracting signals assigned to the reported crystal structure from the observed spectrum, some peaks are remained on the XRD spectrum, therefore, it can be concluded that the sample has two kinds of crystallographic structure (the other structure could not be assigned to a unique structure in the present study). In the case of PCP–TEA, TCP–IMID and 26DCP–2A4MP solids, the XRD spectra suggest that two kinds of crystal are mixed in the solids samples. 

**Figure 6:**
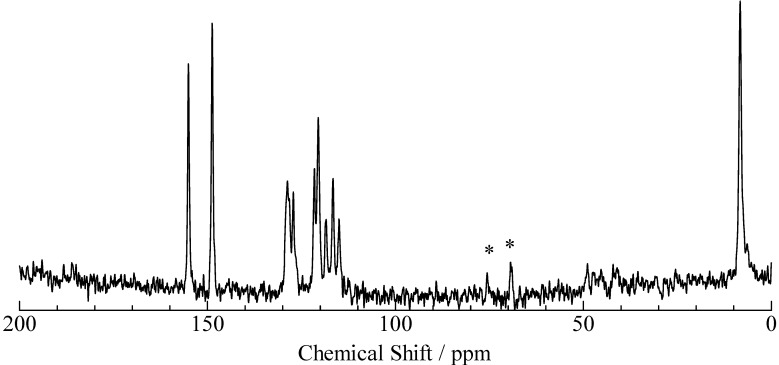
^13^C-CP/MAS-NMR spectrum of pentachlorophenol–triethylamine. The asterisks denote spinning-side-band peaks.

**Figure 7:**
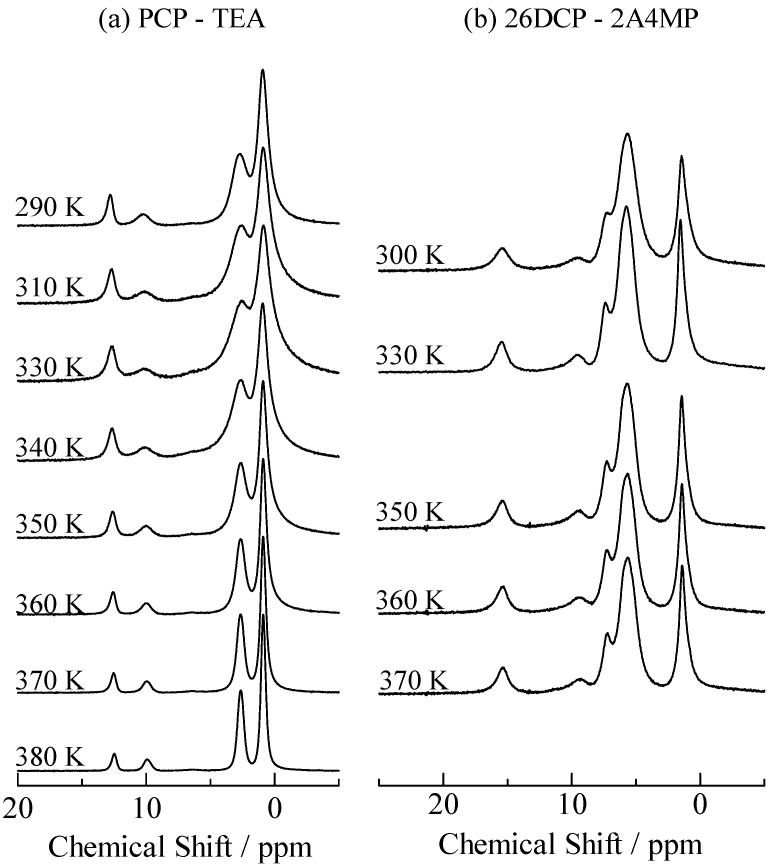
Temperature dependences of ^1^H-MAS-NMR spectra observed in penta-chlorophenol–triethylamine (PCP–TEA) and 2,6-dichlorophenol–2-amino-4-methyl-pyridine (26DCP–2A4MP).

In order to confirm some crystals were mixed in PCP–TEA, ^13^C-CP/MAS-NMR measurements were performed with a MAS ratio of 12 kHz. The spectrum observed in PCP–TEA solids showed nine peaks in a range of 100–170 ppm, as displayed in Figure 6. Since the ^13^C-NMR line was observed at 150.92 MHz with MAS speed of 12 kHz, spinning sidebands could be recorded at 79.5 ppm beside of an isotropic signal. This fact suggests that the signals recorded in the range of 100–170 ppm don’t include any spinning sidebands. Based on the result of ^13^C CS simulation using the same method described above, the peaks observed in the range of 100–170 ppm are assigned to the C atoms in PCP. Since the result of nine peaks recorded on the spectrum is inconsistent with the fact of the number of the C atoms in PCP, it can be concluded that the PCP–TEA sample has more than one H-bond state. In the case of PCP–TEA and 26DCP–2A4MP, ^1^H-MAS-NMR measurements were performed as a function of temperature. Since the spectra show little correlation with temperature as displayed in Figure 7, it can be concluded that no correlation is exists between the two crystal forms in this temperature region. 

### 2.2. H/D Isotope Effects

**Figure 8:**
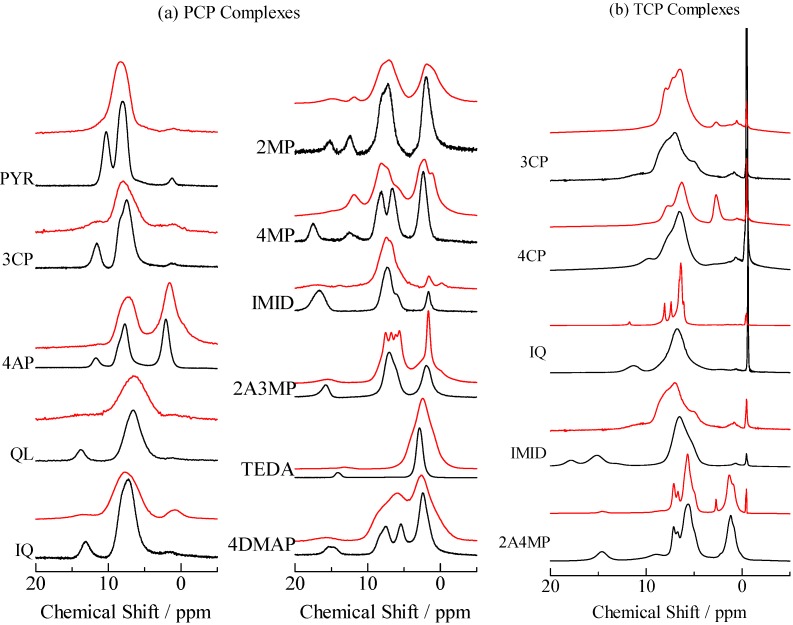
H/D isotope effects on ^1^H-MAS-NMR spectra of (**a**) pentachlorophenol (PCP) and (**b**) 2,4,6-trichlorophenol (TCP) complexes. The NMR signals observed in deuterated and non-deuterated samples are drawn by red and black lines, respectively.

^1^H-MAS-NMR spectra observed in N-Base complexes of PCP and TCP after deuterium substitution are displayed in Figure 8. In order to explain H/D isotope effects on ^1^H-MAS-NMR line-shapes, the ^1^H-MAS-NMR spectra of non-deuterium compounds as shown in Figure 1, Figure 3 are displayed again in Figure 8. Compering these spectra, the peak-intensities assignable to the H atom forming N···H···O type H-bond were reduced by deuterium substitution. These changes suggest that a high ratio of deuterium substitution was achieved in each complex. New peaks were recorded at around 2.5 ppm of TCP–3CP, TCP–4CP, and TCP–2A4MP after deuterium substitution. Since a ^1^H peak of CH_3_CN, which is used for preparation, is generally observed at this frequency, it can be considered that these samples include the solvent. ^1^H-MAS-NMR spectra of some complexes became broad after deuterium substitution, in contrast, narrowing was recorded in PCP-2A3MP-*d* and TCP-IQ-*d*. 

**Figure 9:**
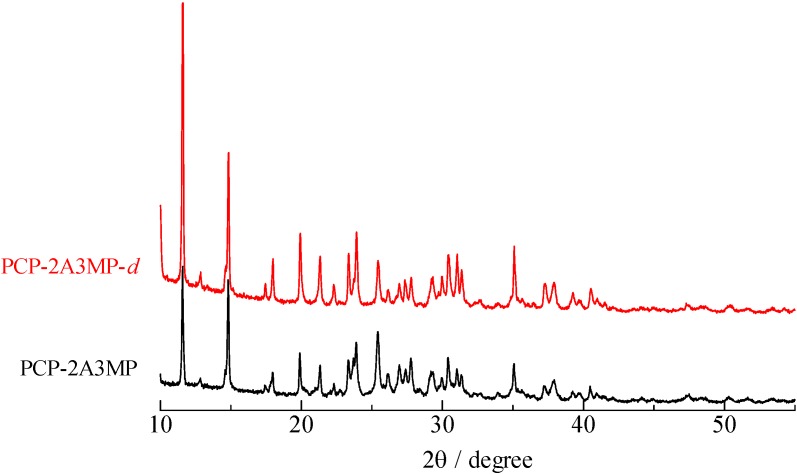
XRD spectra of pentachlorophenol–2-amino-3-methylpyridine.

Since no crystal structures of the complexes have been reported, XRD measurements were performed for both deuterium and non-deuterium complexes. Since the result of XRD observation of TCP-IQ-*d* showed very broad lines, it can be considered that the TCP–IQ crystal is deformed by deuterium substitution,* i.e.*, very sharp signals observed in the ^1^H-MAS-NMR spectrum of TCT–IQ-*d* can be attributed to the spectrum of amorphous states. In the case of PCP–2A3MP, the similar XRD spectrum was obtained before and after deuterium substitution as displayed in Figure 9.

**Figure 10:**
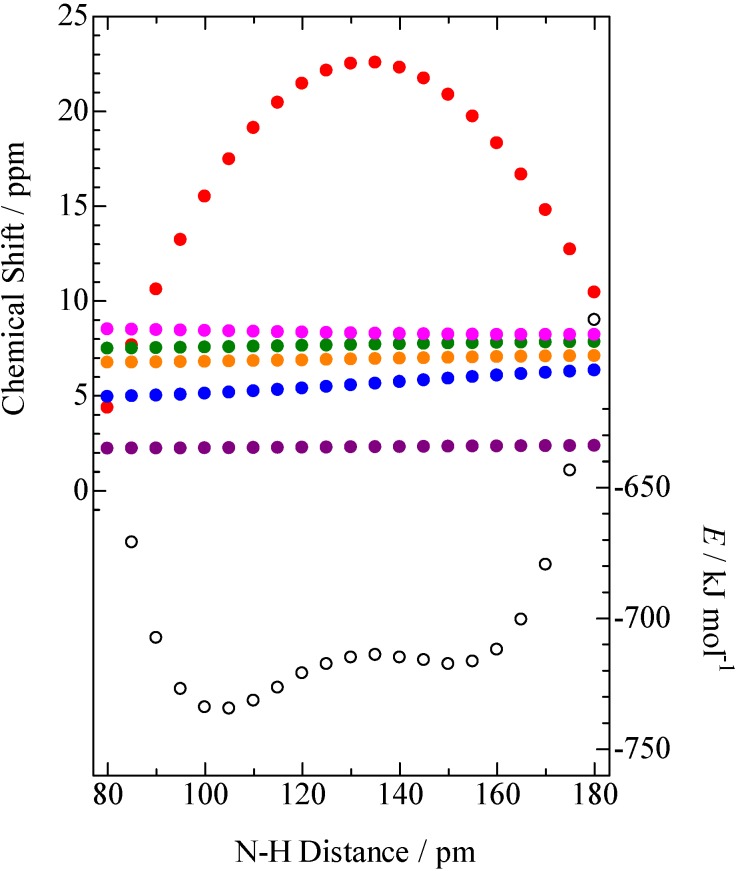
N–H distance dependences of potential energy and ^1^H-NMR chemical shift estimated by B3LYP/6-311+G**; potential energy (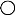), H(NH_2_) (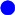), H(CH_3_) (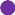), 4-H(*p*) (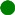), 5-H(*m*) (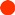), 6-H(*o*) (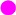), H(N–H···O) (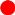).

Therefore, it can be considered that the H/D isotope shifts recorded on the NMR line-shape are caused by the bridging H atom position. In order to discuss origin of H/D isotope effects, DFT simulation were performed. Whole atomic positions of the complex were simulated using a function of B3LYP/6-31+G** in the Gaussian 03 computer program [46] and gave the N–H and H···O distances of 105 and 159 pm, respectively. Shielding tensor calculation was performed with a B3LYP/6-311+G** method. Applying the same simulation to a tetramethylsilane (TMS) molecule, ^1^H CS values were obtained. The ^1^H CS values and potential energies simulated as a function of the N–H distance were plotted in Figure 10. The potential curves showed two minima. The lowest energy was obtained at the N–H length of 105 pm and the second at 150 pm (corresponds to the O–H separation of 114 pm): The simulation showed the probability of finding the bridging H atom near the N atom is higher than that of the O atom side. This estimation is consistent with the fact that the p*K*_a_ value of 2A3MP is larger than that of PCP as shown in Table 1. The CS simulation of the PCP–2A3MP complex showed four peaks in a CS range of 5–9 ppm and one signal at 2.24 ppm. This result was consistent with the number of signals detected on the ^1^H-MAS-NMR spectrum of the deuterated complex as displayed in Figure 8. Based on the result of CS simulation, the peaks were assignable to the H atoms of CH_3_, NH_2_, 5-H, 4-H, and 6-H of 2A3MP molecule, moving from higher to lower fields. The CS simulation showed a tendency that the CS value of NH_2_ (blue circle in Figure 10) is shifted to higher field with decreasing the N–H length. It has been well known that a vibration-energy of an N–H bond becomes low by deuterium substitution, and the N–H length is reduced. Limbach *et al*. have proposed the following relations for H-bonds [33]:

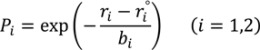
(1)

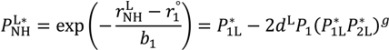
(2a)

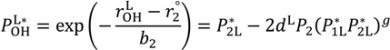
(2b)

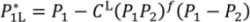
(3a)

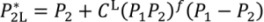
(3b)

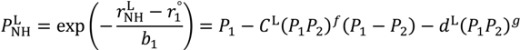
(4a)

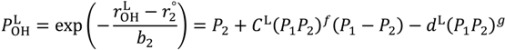
(4b)

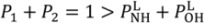
(5)

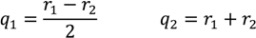
(6)


**Figure 11:**
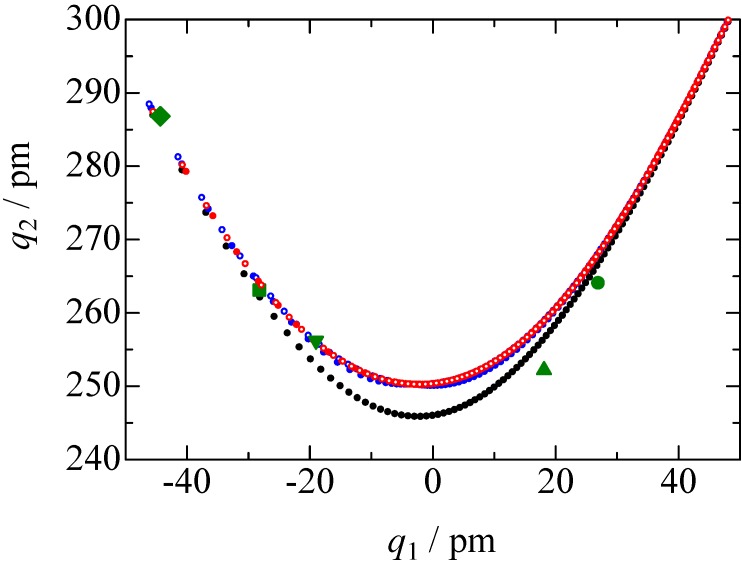
H-bond correlations of O···H···N (*q*_1_ and *q*_2_ are H-bond coordinates defined by equation (6)). The black dots were calculated by eq. (1) and (5) (classical model), blue circles were estimated by equations (1) and (4) (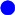
=H) and (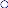
=D) (anharmonic quantum zero point vibration model), and red circles were demonstrated by combination of equation (1) to (3) (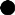
=H) and (○=D) (modified quantum model). The green-colored symbols refer to PCP–2A3MP (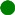), PCP–3CP (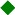), PCP–4DMAP (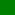), PCP–4MP (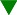), and PCP–TEA (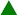).

Here, *P*_1_ and *P*_2_ are bond orders of O–H and N–H, and *r_i_* and 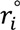 are lengths and equilibrium distances, respectively. The parameter of *b_i_* characterizes the decrease of the bond orders with increasing bond separation. 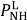 and 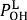 are bond orders corrected by anharmonic quantum zero point vibrational effects, where L = H or D. 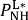 and 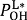 , are modified bond orders of 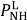 and 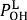 for weak H-bonds. The parameters of *C*^H^ = 360, *C*^D^ = 30, *f *= 5, *d*^H^ = 0.45, *d*^D^ = 0.45, *g* = 2, 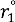
= 94.2 pm, 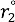
= 99.2 pm, 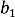
= 37.1 pm, and 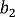
= 38.5 pm have been reported for pyridine – acid complexes [33]. Since Limbach *et al*. explain H-bond characters well employing the above relations and the parameters, the same calculations were performed for some complexes treated in this study. The obtained data are displayed in Figure 11. In the case of PCP–2A3MP, the *q*_1_ and *q*_2_ values were located at an area of weak quantum effects as displayed in Figure 11, therefore, it can be considered that the H/D isotope effects on the ^1^H-MAS-NMR spectrum can be explained by classical quantum models as shown in Figure 10. Based on these results, H/D isotope effects on the ^1^H-MAS-NMR line-shapes of the PCP–2A3MP-*d* complex can be explained by shorting the N–D length as compared with the N–H distance.

## 3. Experimental

Crude crystals were obtained by mixing equimolar amounts of PCP, TCP, 26DCP, 35DCP, and *p*CP, and N-Bases in acetonitrile. The samples were recrystallized by slow evaporation of the CH_3_CN solvent. Some crystals could be prepared, however, some samples showed gel or liquid states at room temperature. Deuterium samples were prepared by the following procedure: PCP was dissolved in NaOH aqueous solution, and the sodium salts were obtained by evaporating H_2_O. By adding the sodium salt into DCl deuterium solution, PCP-*d* crystals were gained. Preparing the deuterated complexes were attempted using the same process described above under N_2_ atmosphere, beginning with PCP-*d* instead of PCP. The same recipe was applied to TCP, 26DCP, 35DCP, and *p*CP for preparing deuterated complexes. Some PCP and TCP crystals could be prepared, however, many complexes were hardly crystallized after deuterium substitution. 

Solid-state high-resolution ^1^H-MAS-NMR experiments were carried out at a Larmor frequency of 600.13 MHz with a Bruker Avance 600 spectrometer. The sample was packed in a ZrO rotor with an outer diameter of 2.5 mm and a spinning rate was kept at 30 kHz through the acquisition of free-induction-decay (FID) signals. Spectra were obtained from FID signals observed after a π/2 pulse. ^1^H CS values were calibrated by external reference of adamantane (δ = 1.91 ppm); in a case of TCP complexes, inner reference of silicon rubber was employed. Recycle time of 5 s was used for normal and deuterium substituted crystals. Sample temperature was controlled by a Bruker VT-3000 variable-temperature unit and estimated from ^207^Pb-NMR chemical sift of Pb(NO_3_)_2_ crystals [47]. 

^13^C-CP/MAS-NMR spectra measurements were carried out at a Larmor frequency of 150.92 MHz with the same spectrometer as ^1^H measurements. The samples were packed in a rotor with an outer diameter of 4.0 mm ZrO rotor. A ramp pulse sequence [48] was employed for recording the spectra with a spinning rate of 12 kHz. The CS of the ^13^C nuclei was calibrated by an external adamantane (*δ* = 29.47 ppm) reference. CP/MAS spectra were recorded with a contact time of 1.0 ms.

XRD powder patterns were obtained using a Bruker D8 ADVANCE equipped with a Cu anticathode. Spectra were recorded using a scan range of 10°–40° with a step angle of 0.02°.

Density-functional-theory (DFT) calculations were carried out using the Gaussian 03 computer program [46] to estimate the potential curve and theoretical values of shielding tensor of ^1^H and ^13^C nuclei.

## 4. Conclusions

CS values of ^1^H nuclei forming hydrogen bonding were observed in phenols (PCP, TCP, 26DCP, 35DCP, and *p*CP)–N-Base complexes by use of solid-state high-resolution ^1^H-MAS-NMR with a MAS speed of 30 kHz. The CS values assigned to the bridging H atoms in these crystals were gradually increased with the p*K*_a_ values of N-Bases in the range of Δp*K*_a_ < 0 (Δp*K*_a_ = p*K*_a_(N-Base) - p*K*_a_(chlorophenols)) and successively decreased in Δp*K*_a_ > 2; the maximum CS values was obtained in the PCP (p*K*_a_ = 5.26)–4MP (6.03), TCP(6.59)–IMID(6.99), 26DCP(7.02)–2A4MP(7.38), 35DCP(8.04)–4DMAP(9.61), and *p*CP(9.47)–4DMAP(9.61) complexes. The result obtained in the PCP complexes is consistent with the inversion point of ^35^Cl NQR frequencies [12,28]. In PCP–4MP, a proton transfer and isotope polymorphism have been reported [12,28,29,30,31,32,33,34,35,36,37,38,39,40]. Since the large ^1^H-NMR CS value of 18.6 ppm was recorded in TCP–IMID as compared with 17.8 ppm of PCP–4MP, it can be expected that the similar properties are obtained in TCP–IMID. Although p*K*_a_ values of N-Bases are determined in aqueous solution, p*K*_a_ dependences of ^1^H-NMR CS values were detected in solids of the complexes. This fact suggests that we can roughly predict electron densities of bridging H atoms in solid samples by comparing p*K*_a_ values of acids and bases. This result can be obtained by ^1^H MAS NMR measurements. In addition, H/D isotope effects on ^1^H-MAS-NMR spectra were detected in PCP–2A3MP. Based on the results of CS simulation using a B3LYP/6-311+G** function, it can be explained that a little changes of the N–H length in H-bond contribute to the H/D isotope shift of the ^1^H-MAS-NMR peaks. 
